# Complete Genome Sequence of Lactococcus garvieae MS210922A, Isolated from Farmed Greater Amberjack (Seriola dumerili) in Japan

**DOI:** 10.1128/mra.00890-22

**Published:** 2022-11-22

**Authors:** Muhammad Akmal, Terutoyo Yoshida, Issei Nishiki

**Affiliations:** a Faculty of Agriculture, University of Miyazaki, Miyazaki, Japan; b Department of Fisheries and Aquaculture, University of Veterinary and Animal Sciences, Lahore, Pakistan; Montana State University

## Abstract

Here, we report the complete genome sequence of a potentially new serotype, Lactococcus garvieae strain MS210922A, isolated from greater amberjack. It is nonreactive to serotype I or II antisera. The complete genome comprises 2,007,159 bp, including 1,929 coding, 16 rRNA, and 58 tRNA genes, with 38.9% G+C content.

## ANNOUNCEMENT

Lactococcus garvieae is a key pathogen infecting freshwater and marine fishes (1). In Japan, two serotypes of L. garvieae, isolated from farmed fish, are known. L. garvieae serotype I has considerably damaged fish farms since 1974 (2). A vaccine containing formalin-inactivated cells dramatically ameliorated the damage caused by serotype I infection. Serotype II was identified in 2012 and has also caused economic losses (3). L. garvieae serotypes I and II can be differentiated using a slide agglutination test with rabbit antiserum and PCR-mediated identification methods (4). However, L. garvieae-like Gram-positive cocci that do not agglutinate with serotype I and II antisera were isolated from diseased greater amberjacks in Japan in September 2021. PCR analysis identified isolate MS210922A as L. garvieae (4). This study aimed to determine the complete genome sequence of a L. garvieae MS210922A strain with a potentially new serotype.

L. garvieae strain MS210922A was isolated from dead farmed greater amberjack brain and cultured on Todd Hewitt agar (Difco, USA) at 25°C for 24 h. A pure culture of MS210922A was suspended in Todd-Hewitt broth containing 10% glycerol and stored at −80°C until use for DNA extraction. Genomic DNA was extracted from MS210922A cultured on Todd-Hewitt agar using the GenElute bacterial genomic DNA kit (Sigma-Aldrich, USA) according to the manufacturer’s protocol and was qualitatively analyzed using a 5200 fragment analyzer system with an Agilent high-sensitivity (HS) genomic DNA 50-kb kit (Agilent Technologies, USA). To eliminate short DNA fragments, the extracted genomic DNA was purified using a short-read eliminator XS (Circulomics, USA). The sequencing library was prepared using the SMRTbell Express template prep kit 2.0 (Pacific Biosciences, USA) following the manufacturer’s protocol and sequenced using the PacBio Sequel IIe instrument. To generate HiFi reads, adapter trimming and computation of consensus sequences were performed using SMRT Link v10.1.0.119528. Consensus sequences with an average quality value of <20 were excluded, resulting in 64,942 HiFi reads with a mean size of 8,748 bp. After excluding HiFi reads below 1,000 bp using Filtlong v0.2.0 (5), *de novo* assembly using Flye v2.9 (6) generated one circular contig. Genome circularity was confirmed by assessing assembly graphs using Bandage v0.8.1 (7), and the assembled genomic data integrity was confirmed using CheckM v1.1.2 (8). The coverage estimated from the total bases used for assembly was 283×. The circularized genome was rotated to the default starting gene, *dnaA*. Functional annotation was performed using the DDBJ Fast Annotation and Submission Tool (DFAST) v1.2.18 (9). The average nucleotide identity (ANI) was calculated using Pyani v0.2.12 (10). Default parameters were used for all software unless otherwise noted.

The chromosomal genome of MS210922A was 2,007,159 bp, with a G+C content of 38.9%. DFAST predicted 2,003 genes, including 1,929 coding, 16 rRNA, and 58 tRNA genes. The ANI values indicated a close association between MS210922A and the serotype I strains, L. garvieae Lg2 (GenBank accession number AP009333) (98.3%) and ATCC 49156 (GenBank accession number AP009332) (98.3%). The 16S rRNA sequences of MS210922A were identical to those of L. garvieae Lg2 and ATCC 49156. Thus, these results identified MS210922A as L. garvieae. The complete genome sequence may help in identifying factors involved in the antigenicity and virulence of L. garvieae isolated from fish.

### Data availability.

The complete genome sequence of Lactococcus garvieae MS210922A has been deposited at DDBJ/EMBL/GenBank under accession numbers PRJDB13734 (BioProject), SAMD00496712 (BioSample), DRR381127 (SRA), and AP026069 (GenBank).
